# Scalable synovial fibroblast sources enable reproducible basal synovial organoid formation: an in vitro platform study

**DOI:** 10.1007/s00296-026-06112-5

**Published:** 2026-05-05

**Authors:** Søren Lomholt, Ann Mai Brøndum Holm Øllgaard, Anni Aagaard Madsen, Morten Aagaard Nielsen, Tue Wenzel Kragstrup

**Affiliations:** 1 Department of Biomedicine, Aarhus University, Høegh-Guldbergsgade 10, bygn. 1115, Aarhus C, 8000 Denmark; 2 Department of Pediatrics, Regional Hospital Viborg, Viborg, Denmark; 3 Department of Rheumatology, Aarhus University Hospital, Aarhus, Denmark; 4 Department of Molecular Medicine, Aarhus University Hospital, Aarhus, Denmark; 5 Department of Rheumatology, Medical Diagnostical Center, University Clinic for Innovative Patient Pathways, Regional Hospital Central Jutland, Silkeborg, Denmark

**Keywords:** Arthritis, rheumatoid, Synovial membrane, Fibroblasts, Endothelial cells, Organoids, Three-dimensional cell culture, Arthritis, experimental, Translational medical research

## Abstract

This study aimed to identify new and scalable sources of cells for forming synovial organoids that align with the pauci-immune synovial pathotype and characterise both structure and cellular organisation of such organoids. The study modified a previously published method for forming synovial organoids using Matrigel, and tested it with combinations of immortalised synovial fibroblasts derived from synovium or synovial fluid together with human umbilical vein endothelial cells or the EA.hy926 cell line. Healthy synovium and synovial fluid derived fibroblasts with human umbilical vein endothelial cells resulted in formation of synovial organoids. Organoid diameter, area and cell count showed no significant differences (*p* > 0.05) in organoids formed with the different fibroblasts. Median CD31 signal intensity was 213 (184–218)) in the vascular-like area, 58 (55–60) in the lining-like area, and 62 (52–72) in the stroma-like area. Median podoplanin signal intensity was 971 (880–1052) in the lining-like area, 341 (310–560) in the vascular-like area, and 342 (281–356) in the stromal-like area. Here, we present novel sources of synovial fibroblasts for successfully forming of pauci-immune-aligned basal synovial organoids. These synovial organoids showed spatial expression patterns of PDPN and CD90 consistent with in vivo synovial fibroblast phenotypes and thereby showed potential as a reproducible basal synovial organoid platform, providing a structural foundation for future mechanistic and translational extensions with additional work needed to establish a fully validated disease model.

**Supplementary Information:**

The online version contains supplementary material available at 10.1007/s00296-026-06112-5.

## Introduction

Immune mediated arthritis is a collective term for diseases such as rheumatoid arthritis (RA), psoriatic arthritis, spondyloarthritis and juvenile idiopathic arthritis. These diseases share several clinical presentations; synovitis, joint pain, enthesitis and recurrent disease flaring, but unfortunately also share the lack of stand-alone diagnostic or treatment prognostic tests. Tests that can shift current treatment regimens from iterative trial-and-error approaches to treatment regimens tailored to the individual patient [1–8]. Recent studies have therefore begun deep phenotyping of the cellular composition of disease-affected tissues, mainly the inflamed synovium. For several types of immune mediated arthritis, studies have shown the presence of both pro-inflammatory synovial fibroblasts, activated endothelial cells, infiltrating immune cells and tissue resident immune cells in the inflamed synovium [9–15]. Cell types that interact and thereby may perpetuate arthritis, as seen in mechanistic studies using complex three-dimensional synovial organoids based on synovial fibroblasts and endothelial cells [16].

One study investigating the RA synovium has, furthermore, opted for a classification of molecular pathotypes, that take into account the internal distribution of different synovial cell types and classifies the synovium into either a fibroblastic pauci-immune, diffuse-myeloid or lympho-myeloid pathotype [17]. Interestingly, these pathotypes correlate with different clinical outcomes, e.g. the pauci-immune pathotype correlate with a lower response rate of anti-tumor necrosis factor treatment compared to the other pathotypes [18]. An important finding since 19–27% of patients in these pathotype-based studies presented with a pauci-immune pathotype [17–19]. The identification of these potential cellular or pathotype targets were also implemented into the first biopsy driven treatment studies in RA. The first of which showed a higher response rate to anti-interleukin 6 receptor versus anti-CD20 treatment in patients who; 1) were intolerant to or failing convention synthetic disease modifying anti rheumatic drug treatment, 2) had failed at least one type of anti-TNF treatment and 3) had biopsies classified as “B-cell poor” [20]. However, subsequent studies using the same synovial B-cell rich/poor stratification of biologic disease modifying anti rheumatic drug naïve patients did not find different treatment responses between anti-CD20, anti-tumor necrosis factor or anti-interleukin 6 receptor treatment [21].

Together, these findings suggest that synovial tissue classifications may have a role in tailoring arthritis treatment to the individual patient. However, synovial biopsies may not be feasible in all clinical settings and, as seen in previous studies, may not effectively stratify patients with arthritis with regards to tailoring individualised treatment. This underlines a still an unmet need for new scalable in vitro tools to supplement synovial tissue classifications with in vitro characterisation of pathogenic pathways and pre-clinical drug testing. Several efforts have been made to create such a tool, e.g. in the form of organoids and organ-on-a-chip, which show promising result but challenges still remain [22]. One of the major challenges for the current models described is the ability to standardise the model to allow for batch-to-batch reproducibility. Additionally, the cost of producing organoids remains high and can limit the usability of these models [22, 23].

We hypothesised that scalable and well-defined sources of synovial fibroblasts, including synovial fluid-derived fibroblasts and healthy tissue-derived fibroblasts, are capable of reproducibly forming basal synovial organoids together with endothelial cells in vitro. We further hypothesised that such organoids would display synovial membrane-like structural organisation and spatial fibroblast phenotypes aligned with a fibroblast-dominant, pauci-immune synovial structural context, thereby providing a reproducible foundation for future mechanistic and translational extensions.

## Materials and methods

Reporting of materials, experimental design, data analysis, and results was guided by the MDAR (Materials, Design, Analysis, and Reporting) framework for transparent reporting of experimental life science research [24].

### Reagents

Full list of reagents used for this study is found under Supplementary materials, Table S1.

### Sources of biological materials

Cells from several biological sources were included for the study. The primary sources of synovial fibroblasts were the commercially available immortalised healthy synovial tissue derived synovial fibroblasts (Innoport, Spain) and an immortalised RA synovial fluid derived synovial fibroblast cell line, HSE [25], that was provided by the Hermann Eibel lab (University of Freiburg, Germany). We also included controls in the form of synovial fluid derived synovial fibroblasts from our biobank samples from patients with rheumatoid arthritis (n = 2) and formalin-fixed paraffin-embedded RA synovial tissue sections (n = 2) was provided by the Deleuran lab (Aarhus University, Denmark.) The included sources of endothelial cells were the immortalised EA.hy926 human umbilical vein endothelial cell line ([26]) and primary HUVECs kindly gifted from the Kalucka Lab (Aarhus University, Denmark).

### Cell isolation and cell cultures

Synovial fibroblasts from our local repository were isolated from synovial fluid of patients with RA. These samples were obtained at the local rheumatology outpatient clinic (Aarhus University Hospital, Denmark) during a disease flare. Synovial fluid mononuclear cells were isolated by density gradient centrifugation with Ficoll-Paque (Sigma-Aldrich, Germany). Synovial fluid derived synovial fibroblasts were grown from the isolated synovial fluid mononuclear cells as previously described [27]. SFMCs were incubated for 48 h in 25 cm^2^ culture flasks (Sarstedt, Denmark) containing DMEM media (Sigma-Aldrich, Germany) supplemented with 10% heat-inactivated fetal calf serum (Sigma-Aldrich, Germany) and 1% penicillin/ streptomycin/ glutamine (Gibco, USA). Cells in suspension were then removed with 1 × PBS wash. Adherent cells were further incubated in supplemented DMEM media (changed every 3–4 days) until > 70% confluent and thus ready for passaging with a trypsin (Gibco, USA)-EDTA (Invitrogen, USA) detachment buffer. At passage three, the cultures were considered to be monocultures of synovial fluid derived synovial fibroblasts.

HUVECs gifted from the Kalucka lab were similarly expanded in culture flasks containing EGM2 media (Lonza, Switzerland) and passaged with trypsin–EDTA until passage three.

Immortalised synovial fluid derived synovial fibroblasts gifted from the Hermann Eibel Lab and the EA.hy926 endothelial cell line were cultured in supplemented DMEM and passaged as described above for primary synovial fibroblasts.

The healthy synovium derived synovial fibroblasts were cultured according to the manufacturer’s recommendation. These were seeded into poly-D-lysine (Gibco, USA) coated culture flasks and cultured in Synoviocyte Medium Plus (Innoprot, Spain) and passaged as described above.

All cell types were cultured at 5% CO_2_, 37 °C and 85% relative humidity. When necessary, cells were frozen for storage in DMEM media with 20% fetal calf serum and 10% DMSO at -140 degrees Celsius.

Cell lines were routinely mycoplasma tested to ensure standard quality control procedures and were used between in-house passages 3–7 to limit potential phenotypic drift from previous characterisation [27, 28].

### Synovial organoid protocol

The synovial organoid protocol used for this study was modified from previously published studies of specific pathogenesis mechanisms in RA [16, 29]. The synovial organoids were formed from a combination of 100.000 synovial fibroblasts and 100.000 endothelial cells. The cells were harvested from 75 cm^2^ culture flasks (Sarstedt, Denmark) and spun down in 15 mL tubes (Sarstedt, Denmark). Supernatant was aspirated, on ice cells were mixed well in extracellular matrix gel, Matrigel (Corning, USA, lot: 3,121,003), and plated in 35 μl droplets on poly-HEMA (Sigma-Aldrich, Germany) coated 12w plates (Sarstedt, Denmark). The synovial organoids were then cultured in EGM2 media or supplemented DMEM media for 21 days with media change every 3–4 days. After which, the synovial organoids were manually isolated with wide-bore tips, fixed in 4% paraformaldehyde (Sigma-Aldrich, Germany) and transferred to the local pathology department (Aarhus University Hospital, Denmark) for paraffin embedding, sectioning onto glass slides and standard haematoxylin/eosin (HE) staining using their standardised in-house protocol.

### Immunofluorescence (IF) staining

Synovial organoid and synovial membrane sections were heated at 37 degrees Celsius overnight before clearing in 3 × 5 min xylene and rehydration in 2 × 5 min of decreasing ethanol concentrations (100%, 96% and 70%) concluding with 5 min incubation in PBS. Epitopes were retrieved in a boiling water bath while incubated in the citrate-based, pH 6.0, Target Retrieval Solution (Agilent, USA) for 25 min. Slides were then left on the tabletop to cool in retrieval buffer for 20 min. Samples were blocked with 10% normal goat serum (Invitrogen, USA) for 25 min at room temperature (RT), washed × 3 with PBS and afterwards incubated with primary antibodies overnight at 4 degrees Celsius. Slides were washed × 3 in PBS after primary antibody incubation and then incubated with secondary antibodies for 30 min, in the dark and at RT. Following a final × 3 PBS wash, slides were coverslipped with the DAPI containing ProLong™ Gold Antifade Mountant (Invitrogen, USA) and kept at 4 degrees Celsius in the dark until imaging.

The following primary antibodies were used for experiments; rat anti-human Podoplanin (PDPN.

) (clone: NZ1.3, Thermo Fisher, USA), rabbit anti-human CD90 (clone: EPR3132, Abcam,USA), mouse anti-human CD31 (clone: JC/70A, Thermo Fisher, USA). Secondary antibodies were goat anti-rat Alexa Fluor 555, goat-anti-rabbit Alexa Fluor 647, goat anti-mouse DyLight 755 (all from Thermo Fisher, USA). Staining controls included isotype controls and single stains.

### Fluorescence microscopy

HE stained and IF stained samples were imaged on an upright brightfield and widefield fluorescence microscope (VS120, Olympus, Japan), with an AVT Pike F-505C VC50 progressive scan CCD color camera, 2x-40 × objectives and single-band emitters the following classical channels; Hoechst, FITC, Cy3, Cy5 and Cy7. Acquisition was done with the vendors VS-ASW software. HE stained slides were imaged at 20 × using the standard settings. Exposure times for IF stained samples were optimized to utilise as close to 80% of the dynamics range of the detector without introducing unacceptable noise.

### Image visualisation, structural measurements, and digital segmentation

Images were visualized and analysed with the free open-source software QuPath (v. 0.5.1.) [30]. Initial quality control of images was performed to identify any unacceptable issues with focus, tissue folding and unspecific staining. Samples with unacceptable issues were excluded from the following digital cell segmentation and analysis.

The tissue area of HE stained organoids were identified with the “simple tissue detection” function in QuPath and tissue diameters were measured with the “Add shape feature” function. Lining layer thickness was obtained from the average of 10 manual measurements of the lining layer-like area on the periphery of the HE stained organoids.

Cell segmentation was done with QuPath's “detect cells” algorithm. In short, the algorithm identifies cell nuclei based on nuclear staining (haematoxylin for HE images and DAPI for IF images), the user determined settings of haematoxylin/DAPI threshold, what radius to assign each cell, maximum and minimum area of a nucleus. Cell segmentation workflow was: 1) Annotate all regions of interest using QuPath’s drawing tools or “simple tissue detection” function. 2) Optimize “detect cell” settings to avoid misclassifications of bundled cells etc., and for IF images only; 3) Extract and trim imaging data to include IF signal from the appropriate cell compartments (e.g. nuclear staining for DAPI and cytoplasmic staining for surface markers).

The artificial neural network algorithm from QuPath’s “train pixel classifier” was used to identify and annotate lining layer-like and vascular-like areas of IF stained organoids. Image sections from several synovial organoids of the same batch were manually annotated to train the classifier algorithm before it was applied to the full-size images of all organoids within that batch. All detected cells within a classified area were assigned the corresponding area label while cells not in either were classified as stromal area cells.

In all figures n refers to the number of independent organoids used for analyses.

### Statistics

Physical measurements and IF signal were quantitatively visualised and analysed with Prism (v. 10, GraphPad Software, USA). IF imaging data was furthermore visualised with the base UMAP function for R (v. 4.3.2) in RStudio (v. 4.0.735) [31, 32]. Between-group comparisons for structural measurements were tested using the Mann–Whitney test because data were not normally distributed. Immunofluorescence intensity data in Fig. 5 are presented descriptively. P < 0.05 was considered significant. Coefficient of variation (CV) was calculated as the standard deviation divided by the mean.

## Results

### Healthy tissue derived and synovial fluid derived synovial fibroblasts form synovial organoids together with HUVECs

**Fig. 1:**
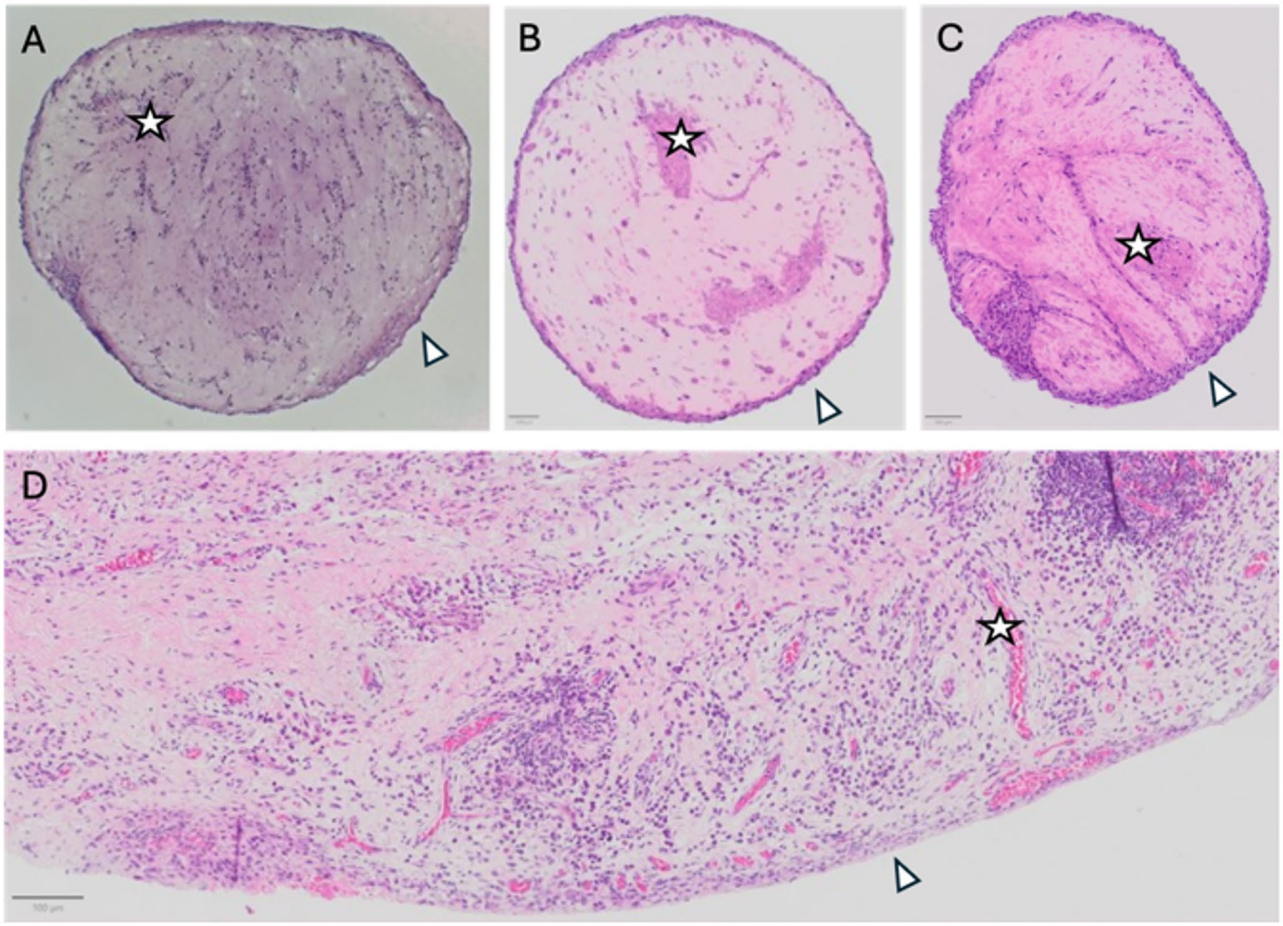
**A**-**C** Representative haematoxylin and eosin staining of synovial organoids formed by HUVECs and: **A** primary synovial fluid derived synovial fibroblasts, **B** immortalised synovial fluid derived synovial fibroblasts, and **C** immortalised healthy synovium derived synovial fibroblasts. **D** Haematoxylin and eosin staining of a synovial membrane for comparison. Arrowheads: Lining-like area, Stars: Vascular-like areas. Scale bars indicate 100μm

We initially tested whether any of our included synovial fibroblasts were able to form synovial organoids together with HUVECs. As presented in Fig. 1A–C, both primary synovial fluid derived synovial fibroblasts, along with immortalised synovial fibroblasts derived from synovial fluid and healthy synovium were able to form organised structures together with HUVECs. Furthermore, all structures showed a cell dense lining-like periphery (Fig. 1A–C, arrowheads) and deeper cell-dense areas suggestive of vascular-like endothelial regions (Fig. 1A–C, stars) similar to what was seen in the synovial membrane, Fig. 1A–D. Based on this we considered immortalised synovial fibroblasts from both synovial fluid and healthy synovium as novel candidate sources of synovial fibroblasts and proceeded with further characterisation of both.

### Structural measurements of successfully formed synovial organoids

**Fig. 2:**
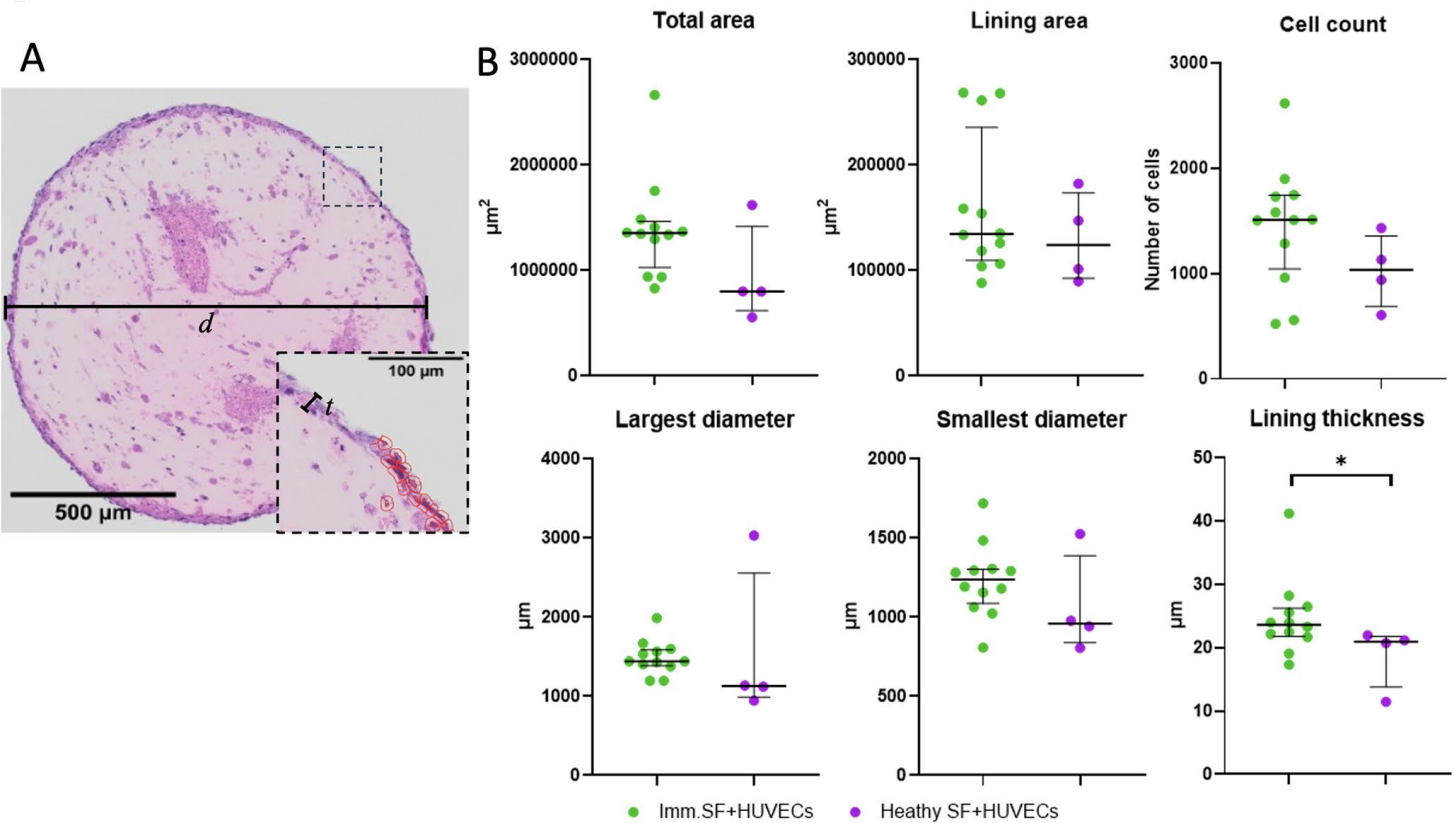
Structural and cellular measurements of synovial organoids formed with immortalised synovial fluid derived fibroblasts or healthy synovium derived synovial fibroblasts together with human umbilical vein endothelial cells. A) Representative image of a synovial organoid showing how structural measurements d (diameter), t (lining layer thickness), and identified cells (red overlaid masks). B) Dot plot of structural measurements with median and interquartiles. Immortalized synovial fluid derived synovial fibroblasts + HUVECs n = 12, Healthy synovium derived synovial fibroblasts + HUVECs n = 4. Each dot represents an independent organoid. *p < 0.05. Abbreviations: Immortalised synovial fluid derived synovial fibroblasts: imm.SF, healthy synovium derived synovial fibroblasts: Healthy FS, human umbilical vein endothelial cells: HUVECs

After identifying that both sources of immortalised synovial fibroblasts were able to form synovial organoids with HUVECs we next evaluated whether they formed similar structural organisation. In general, we did not find that the two types of synovial organoids differed significantly, except in the manually measured lining-layer thickness, Fig. 2.

We, furthermore, calculated the intra-batch CV for each of the measurements to explore whether any of the measurements could be potential markers of analysis, should the model be used for in vitro testing. In synovial organoids based on immortalised synovial fibroblasts we found two potential markers for structural measurements: The largest diameter with a CV of 18.6% and smallest diameter with a CV of 14.3%. CVs were not calculated for synovial organoids formed from healthy synovium derived synovial fibroblasts because of the lower number of samples in the analysis.

### The source of endothelial cells for synovial organoids matters

**Fig. 3:**
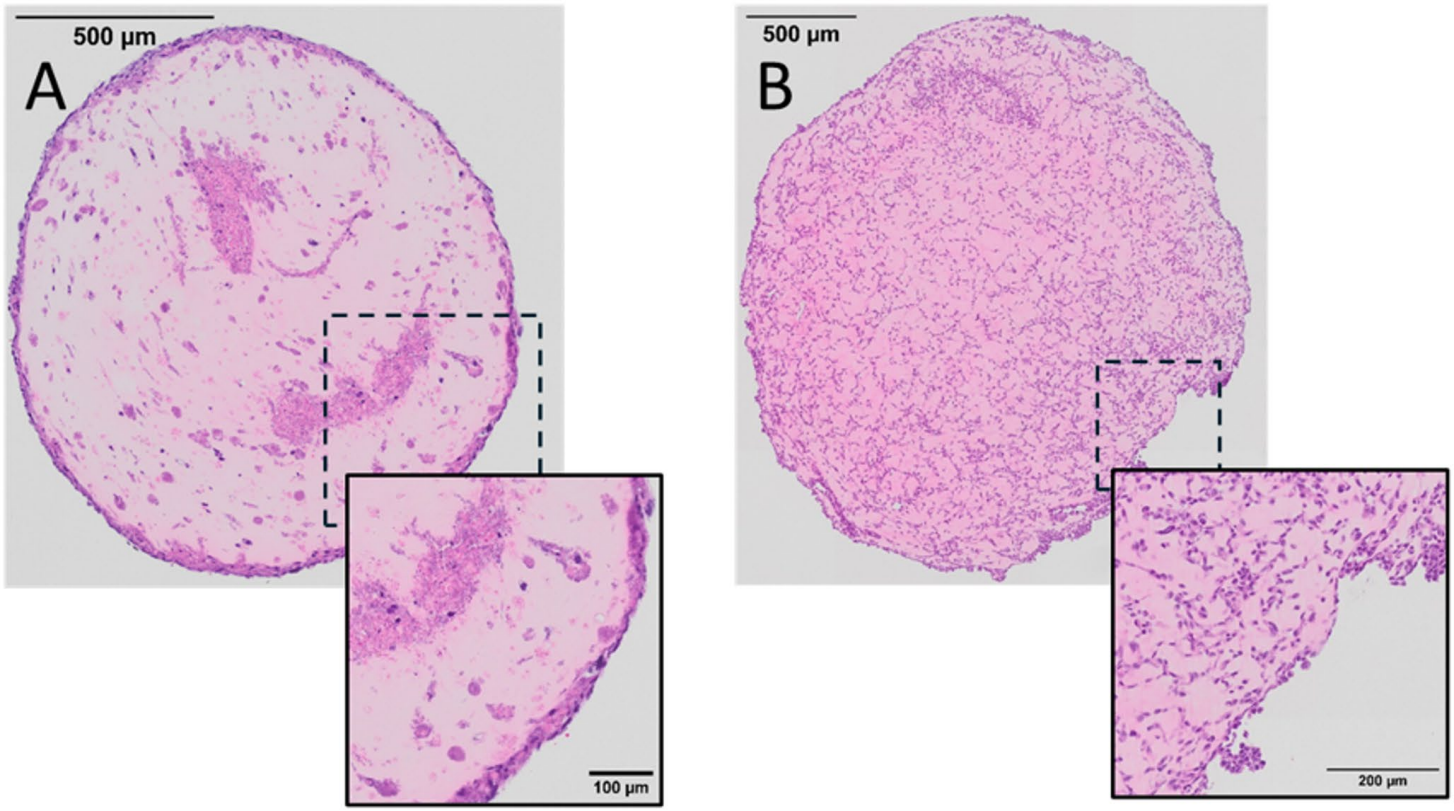
Clear structural differences between synovial organoids formed with different sources of endothelial cells. **A** Representative image of an immortalised synovial fibroblast and HUVEC synovial organoid. **B** Representative image of an immortalised synovial fluid and EA.hy926 synovial organoid

Following initial structural evaluation of synovial organoids formed with the novel candidate sources of synovial fibroblasts, we tested whether the immortalised EA.hy926 cell line was a viable and potentially more standardised source of endothelial cells. We found that synovial organoids formed with the EA.hy926 cell line were distinctly different from our previous synovial organoids, Fig. 3. Specifically, we were unable to visually identify a lining layer periphery and found that cell density was more uniform than in synovial organoids formed with immortalised synovial fibroblasts and HUVECs. The same pattern for cellular distribution and additionally, an increased rate of fragmentation were observed in synovial organoids formed with healthy synovium derived synovial fibroblasts and EA.hy926, supplementary Figure S1.

### Choice of culturing media affects formation of synovial organoids

**Fig. 4:**
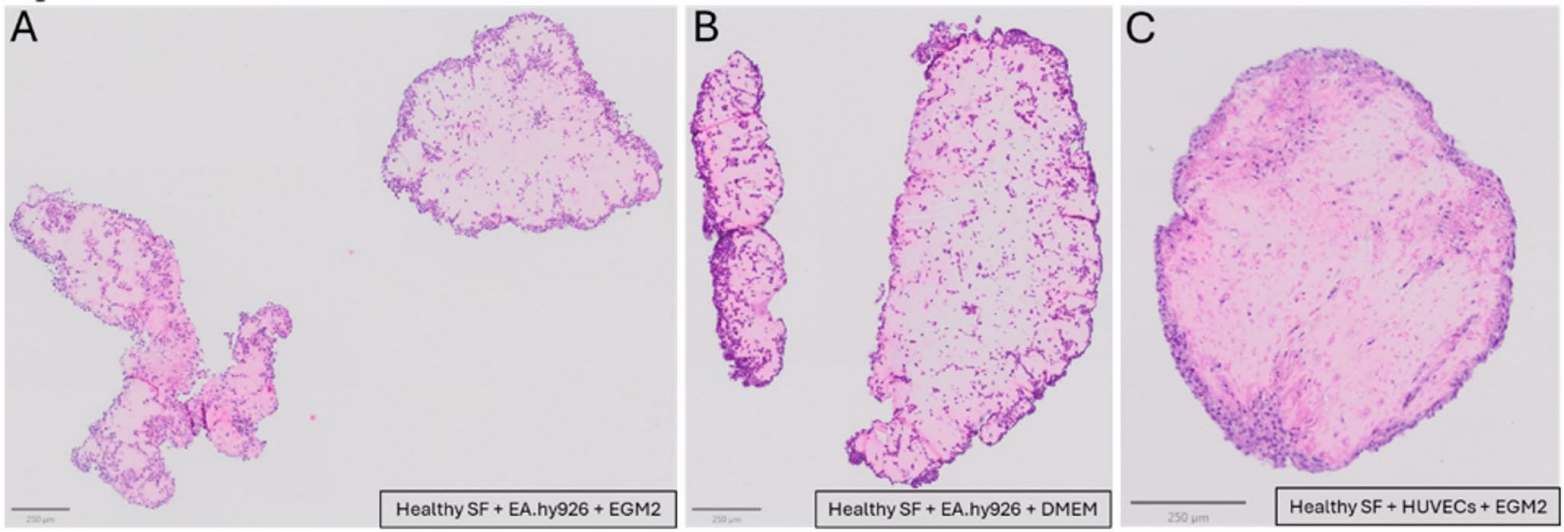
Structural differences in synovial organoids formed with different sources of endothelial cells and cultured in supplemented DMEM media and EGM2 media. No organoids formed from the combination of healthy synovium derived synovial fibroblasts, human umbilical vein endothelial cells and supplemented DMEM media. **A** Fragmented organoid formed with healthy synovium derived synovial fibroblasts and EA.hy926 cells in EGM2 media. **B** Fragmented organoid formed with healthy synovium derived synovial fibroblasts and EA.hy926 cells in supplemented DMEM media. **C** Organoid formed with healthy synovium derived synovial fibroblast and HUVECs in EGM2 media. All scale bars represent 250 μm. *SF* Synovial fibroblasts, *HUVECs* Human umbilical vein endothelial cells

To optimize running costs for synovial organoid production, we tested whether EGM2 media could be exchanged with a less expensive supplemented DMEM media, which was used for all of the non-HUVECs monocultures. We chose to test this on healthy synovium derived synovial fibroblasts in combination with HUVECs or EA.hy926 cells. This also allowed us to test whether supplemented DMEM media could positively affect the structure of synovial organoids formed with healthy synovium derived synovial fibroblasts and the EA.hy926 cell line. As described above, we found that organoids form with combinations of EA.hy926 cells showed increased fragmentation regardless of the type of media used, Fig. 4A, B. We, furthermore, found that no synovial organoids formed when healthy synovium derived synovial fibroblasts and HUVECs were cultured in supplemented DMEM, supplementary Figure S2.

### Digital segmentation of synovial organoids identifies distinct lining-like and vascular-like areas

**Fig. 5:**
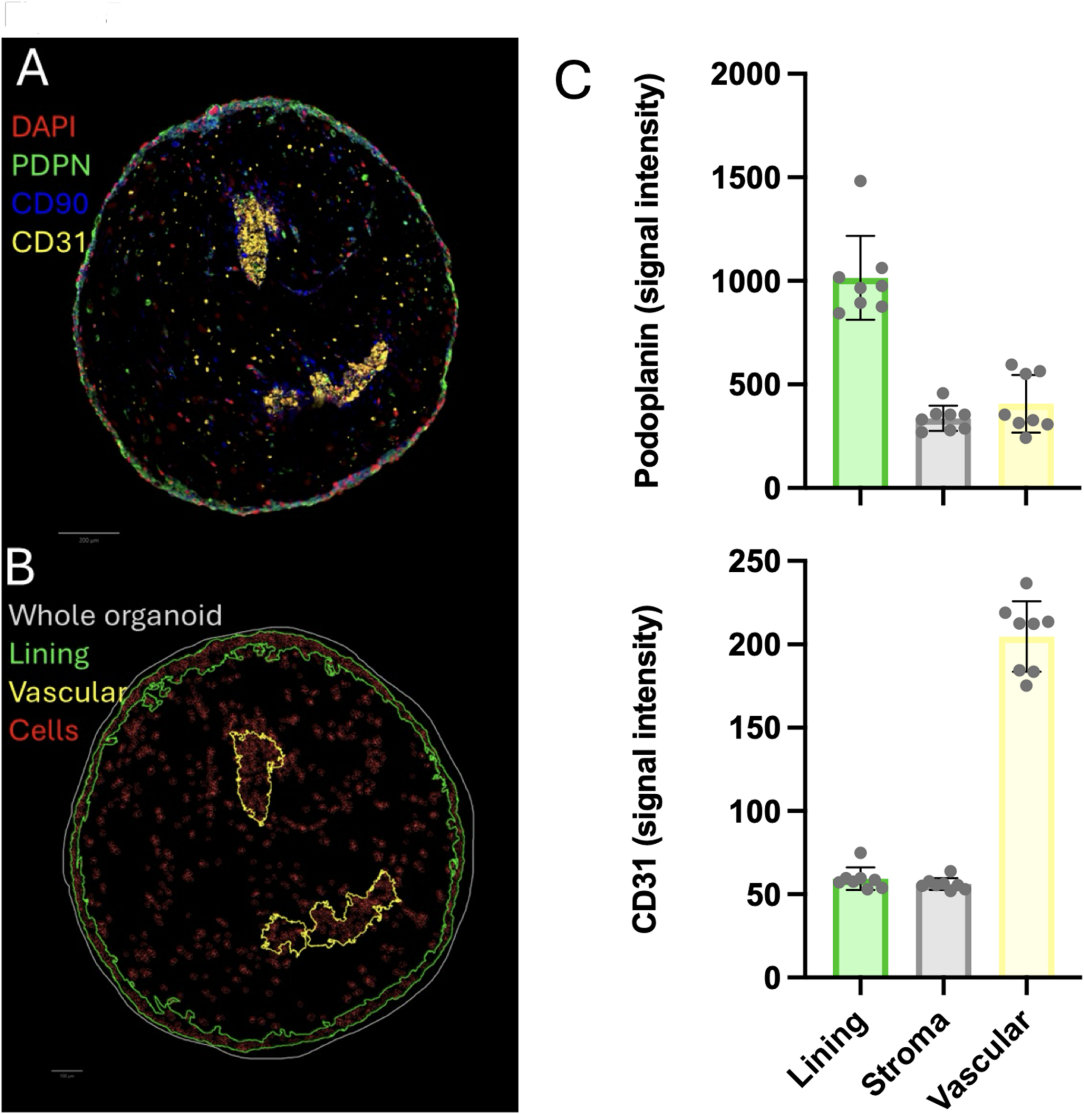
Distribution of the cell markers CD31, CD90 and podoplanin in synovial organoids. **A** Representable immunofluorescent staining of an immortalised synovial fibroblast + HUVEC synovial organoid. **B** Immunofluorescence signal-based segmentation and annotations of lining and vascular (endothelial) areas, along with segmented cells (red masks). **C** Immunofluorescence staining intensity of cells plotted against the segmented area of synovial organoids formed with immortalised synovial fibroblast + HUVECs (n = 8). Stroma area is defined as whole tissue minus Vascular and Lining annotations. Colour visualise the segmented areas. Abbreviations: Synovial fibroblast: SF, Human umbilical vein endothelial cells: HUVECs, Podoplanin: PDPN

Next, we turned to further characterisation of the cellular distribution and synovial fibroblast expression of phenotypical markers. Digital segmentation of synovial organoid areas and cells showed that organoids formed with both novel candidate sources of synovial fibroblasts (synovial fluid and healthy synovial tissue) presented with distinct areas of primarily CD31 and podoplanin expression (Fig. 5A and B).

In Fig. 5C, organoids formed with immortalised synovial fibroblasts + HUVECs were used to descriptively show signal intensity of CD31 and podoplanin in lining layer, vascular and stromal areas. Median CD31 signal intensity was 213 (184–218)) in the vascular-like area, 58 (55–60) in the lining-like area, and 62 (52–72) in the stroma-like area. Median podoplanin signal intensity was 971 (880–1052) in the lining-like area, 341 (310–560) in the vascular-like area, and 342 (281–356) in the stromal-like area. (Fig. 5C).

## Discussion

Our study presents two novel biological sources of synovial fibroblasts for forming a pauci-immune-aligned basal synovial organoids: immortalised synovial fluid derived synovial fibroblasts from a patient with RA and commercially available immortalised healthy synovium derived synovial fibroblasts. Both novel sources of synovial fibroblasts were successful in forming synovial organoids when combined with HUVECs. Furthermore, these two scalable sources of synovial fibroblasts both showed distinct internal cellular organisation similar to what is found in the synovial membrane. Specifically, we found that synovial fibroblasts in the lining-like area expressed podoplanin while cells in the vascular area expressed CD31 [11, 33]. Accordingly, the present study establishes and structurally characterises a reproducible basal synovial organoid platform. However, functional, transcriptomic and translational validation remain necessary future steps.

The idea of three-dimensional cultures for investigating arthritis pathology is not new [16, 29, 34–36]. Early in vitro models sought to move beyond direct culture of joint biopsy specimens. However, they largely failed to recapitulate the structural and cellular complexity of the synovium [37]. Over time, several models with different proposed uses have been published, ranging from synovial fibroblast monocultures for assessing tissue remodelling to synovial fibroblasts, endothelial and macrophage spheroid cultures for in vitro investigations of signalling pathways and drug testing [34, 36]. The published models also differ on the scaffold used for the cultures. While some scaffolds used are mixes of single components, such as methylcellulose and collagens, others, including ours, are a mixture of several basal membrane and extracellular components combined in extracellular matrix gels [16, 35, 36]. Direct comparison between these models is thus difficult due to the considerable variation in cell types and reagents used to form them.

We do, however, argue that three-dimensional arthritis models should strive to not only be a mixture of the cells and matrix constituents of the synovial membrane. It should also mimic the cellular organisation and the cellular phenotypes of synovium, like the NOTCH3 spatial identity signal from endothelial cells in the synovial organoid model from which our study adapted its protocol [16].

As mentioned above, we found that both immortalised healthy synovium derived synovial fibroblasts and immortalised synovial fluid-derived synovial fibroblasts successfully formed synovial organoids together with HUVECs. Interestingly, our analysis was also able to detect different in vivo-like synovial fibroblast phenotypical traits based on digital segmentation of immunofluorescence images. Our study specifically found that synovial fibroblast in proximity to endothelial cells showed less podoplanin expression but maintained their expected CD90 expression [11, 27]. Interestingly, several studies have identified a sub-lining/perivascular CD90 + synovial fibroblast subset with a pro-inflammatory phenotype [9, 11, 38]. Findings that together suggest that synovial fibroblasts in our basal synovial organoid model not only mimic the synovial lining layer but also the perivascular/sub-lining area. The model was therefore termed “pauci-immune-aligned” to convey the emphasis that this model is dominated by synovial fibroblasts and endothelial cells, that resemble several characteristics of the immune cell poor arthritis synovium, but does not include a CD68 + lining layer resident macrophages [17]. This model is based on cellular composition and spatial organisation rather than transcriptomic or molecular pathotype validation. Accordingly, the model should be interpreted as a basal platform aligned with fibroblast-dominant synovial contexts, rather than as a direct in vitro representation of a clinically defined pauci-immune synovial pathotype. The absence of immune cells and transcriptomic benchmarking represents an important limitation of the current study.

We consider these novel synovial fibroblast sources a useful step towards more scalable synovial organoid models. Both sources supported organoid formation with HUVECs, and the workflow for organoid generation and image-based structural characterisation was feasible without highly specialised equipment. In that sense, the model may provide a practical basal platform for future in vitro studies that require reproducible and accessible synovial organoid generation. However, its translational relevance remains to be established through additional functional validation and extension with other relevant cell types.

Because of this we expect this scalable basal synovial organoid model has the potential to evolve with future needs of in vitro arthritis research. We especially expect that the addition of immune cells would significantly increase the potential applications of the model. Such a strategy could also increase alignment with synovial tissue classification by optimising the protocol towards addition of lining-layer-like macrophages, B-cell containing lymphoid follicles or inclusion of patient derived peripheral immune cells to reproduce an in vivo-like infiltration of immune cells. Other types of additions could be to include cell-free synovial fluid to resemble the local pro-inflammatory cytokine milieu of the joint, or inclusion of specific chemokine/cytokine cocktails that stimulate known inflammatory processes.

Based on these additions, the modified model could potentially serve several in vitro purposes and goals; (1) Translational in vitro investigations of activated signaling pathways associated with arthritis, (2) Validation of novel drug targets by examining the effect of both targeted and down-stream signal pathways, (3) Reduction in the use of animal models for pre-clinical drug testing, etc.

In general, our study benefits strongly from testing several combinations of cell types and sources to identify which are successful in forming synovial organoids. We, furthermore, regard it as a strength that the majority of our image data were segmented and extracted with the widely used and published tool, QuPath. This allowed the study to move towards a more unbiased and quantitative approach to image analysis of both HE stained and IF stained samples. The study also benefits from using both staining techniques on adjacent sections of the synovial organoids, which made it easier to compare information from the two.

The study does, however, also have limitations. The number of independent biological replicates in parts of this study is a limitation as it limited the statistical power and generalisability of the findings. The synovial organoid model presented here is limited by only mimicking the in vivo interaction of synovial fibroblasts and endothelial cells in the arthritis synovium. A key limitation of the present study is the absence of functional readouts, such as transcriptomics, inflammatory stimulation or downstream molecular responses, which would be required to directly assess disease-relevant activity or translational responsiveness. The study was intentionally designed to establish and characterise a reproducible basal synovial organoid platform, and evaluation of functional outputs, drug target validation, or predictive translational performance should therefore be considered prospective rather than demonstrated. As outlined above, we also expect that further addition of patient derived cells or specific in vitro derived cell types are needed for this pauci-immune-aligned basal synovial organoid model to reach its full potential as an in vitro tool for investigating immune mediated arthritis.

## Conclusion

Our study has identified several novel sources of synovial fibroblasts, that successfully form synovial organoids together with HUVECs. These pauci-immune-aligned basal synovial organoid models present with synovial membrane-like structural organisation and synovial fibroblast expression profiles that mimics synovial fibroblast expression of CD90 and podoplanin in the synovial membrane. In conclusion, this study supports the feasibility of a reproducible basal synovial organoid platform and characterises its structural and phenotypic features, serving as a foundation for subsequent functional and translational studies in efforts to improve the translatability of the model.

## Supplementary Information

Below is the link to the electronic supplementary material.

Supplementary Material 1
