# Supplementary material for: Scalable synovial fibroblast sources enable reproducible basal synovial organoid formation: an in vitro platform study
Source: Rheumatol Int. 2026 May 5;46(5):83. doi: 10.1007/s00296-026-06112-5 (PMC13139273; doi:10.1007/s00296-026-06112-5)
Supplement: Supplementary file 1 — Supplementary Material 1 [file 296_2026_6112_MOESM1_ESM.docx]

**Supplemental materials**

Table S1: Commercially available materials

| **Reagents** | **Vendor** | **Cat #** |
| --- | --- | --- |
| *Cell lines* |  |  |
| Healthy tissue derived synovial fibroblasts | Innoprot | P10972-IM |
|  |  |  |
|  |  |  |
| *Buffers, media and solutions* |  |  |
| PBS | Biowest | L0615 |
| DMEM | Sigma-Aldrich | D8437 |
| Fetal calf serum | Sigma-Aldrich | F9665 |
| EGM2-Plus BK media | Lonza | CC-5035 |
| Formaldehyde | Sigma-Aldrich | F1635 |
| Pen-strep-glutamine | Gibco | 10378-016 |
| Matrigel | Corning | 356231 |
| Poly-HEMA | Sigma-Aldrich | P3932 |
| Ficoll Paque | Sigma-Aldrich | GE17-1440-02 |
| Trypsin | Gibco | 15090-046 |
| EDTA | Invitrogen | 15575-038 |
| Normal goat serum | Invitrogen | 31872 |
| Synoviocyte Medium Plus | Innoprot | P60127-Plus |
| Poly-D-lysine | Gibco | A38904-01 |
| DMSO | Sigma-Aldrich | D8418 |
| Citrate-based, pH 6.0, Target Retrieval Solution | Agilent | S236984-2 |
| ProLong^TM^ Gold Antifade Mountant | Invitrogen | P10144 |
|  |  |  |
|  |  |  |
| *Utilities* |  |  |
| 15 mL tubes | Sarstedt | 62.554.502 |
| 12 well plates | Sarstedt | 83.3921 |
| 25 cm^2 cell culture flasks | Sarstedt | 83.3910.002 |
| 75 cm^2 cell culture flasks | Sarstedt | 83.3911.002 |


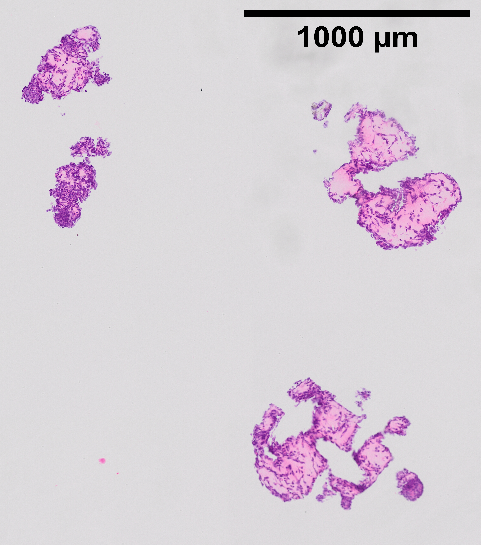

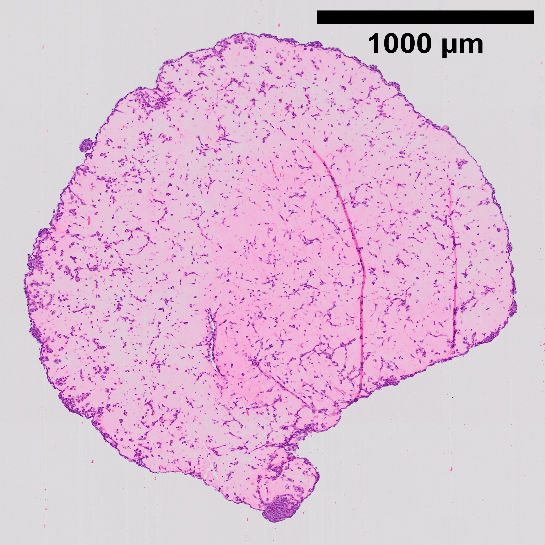

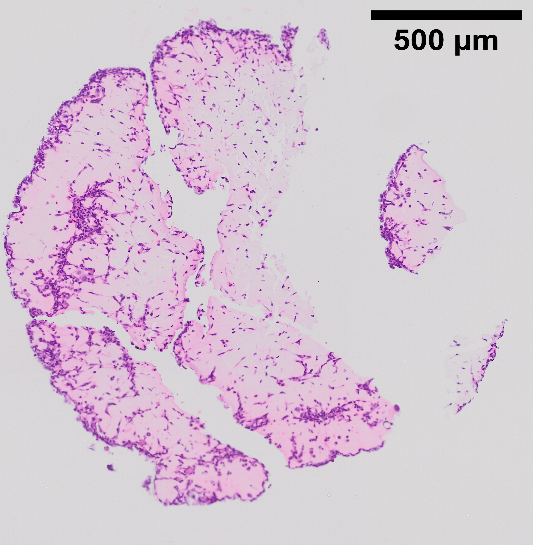


Figure S1: Cellular distribution and fragmentation in synovial organoids consisting of healthy synovium derived synovial fibroblasts and EA.hy926


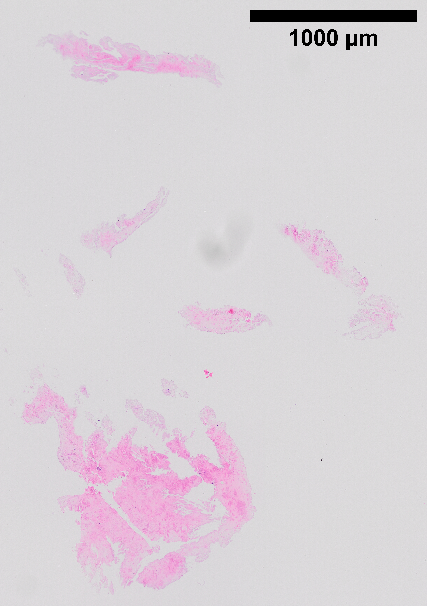

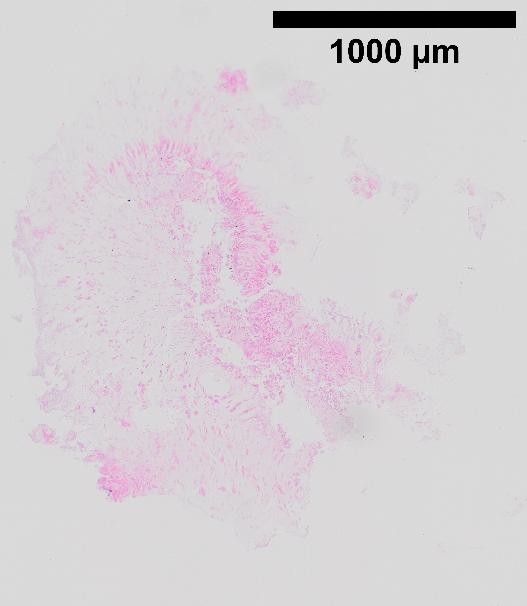


Figure S2: Lack of synovial organoids when healthy synovium derived synovial fibroblasts and HUVECs were cultured in supplemented DMEM
